# The multifaceted role of quaking protein in neuropsychiatric disorders and tumor progression

**DOI:** 10.3389/fnins.2024.1341114

**Published:** 2024-10-16

**Authors:** Zeshang Guo, Bo Liu, Ying Wei, HeFei Wang, Qingquan Zhang, Xinyu Hong

**Affiliations:** ^1^Department of Neurosurgery, The First Bethune Hospital of Jilin University, Changchun, Jilin, China; ^2^Department of Neurosurgery, Cangzhou Central Hospital, Cangzhou, Hebei, China; ^3^Department of Radiology, The First Bethune Hospital of Jilin University, Changchun, Jilin, China; ^4^Cancer Center, First Bethune Hospital of Jilin University, Changchun, Jilin, China; ^5^Department of Pharmacy, Quanzhou First Hospital Affiliated to Fujian Medical University, Quanzhou, Fujian Province, China

**Keywords:** quaking protein, neuropsychiatric disorders, tumor progression, non-coding RNA, glioblastoma multiforme

## Abstract

The Quaking protein (QKI) belongs to the STAR protein family and plays a significant role in the development of the nervous system. It serves as a crucial regulator in the processes of tumor progression and cardiovascular system development. Within the central nervous system, QKI has been associated with the onset and progression of numerous neuropsychiatric disorders, including schizophrenia, depression, ataxia, and Alzheimer’s disease. In malignant tumors, the methylation of the QKI promoter inhibits its expression. QKI primarily involves in the generation, stability, and selective splicing of non-coding RNA, as well as in mRNA translation. The role of QKI in the tumor microenvironment should not be overlooked. Especially in Glioblastoma Multiforme (GBM), although QKI is not the primary mutation, it still plays a vital role in maintaining the stemness of GBM. However, the mechanisms and further studies on this topic demand extensive basic and clinical trials.

## 1 Overview of QKI

The Quaking gene encodes the QKI protein, a member of the STAR (signal transduction and activation of RNA) family of RNA-binding proteins with KH domains, also known as GSG (GRP33, Sam68, and GLD-1) or SGQ (Sam68, GLD-1, and Qk1). The genes encoding QKI proteins are located on chromosome 6 in humans and chromosome 17 in mice. The Quaking gene spans approximately 65 kb and contains 9 exons. Through alternative splicing, it can produce 5 different mRNA isoforms that share the first four exons (Kondo et al., 1999). The three primary alternatively spliced mRNAs of the Quaking gene, measuring 5, 6, and 7 kb, encode the QKI-5, QKI-6, and QKI-7 proteins, respectively. These proteins differ in the last 30 amino acids at their C-terminus (Ebersole et al., 1996). They also differ in cellular localization: QKI-5 is found in the nucleus and regulates the alternative splicing of myelin-associated glycoprotein (Scott et al., 2004) and the nuclear retention of myelin basic protein mRNA (Wu et al., 2002). QKI-6 is present in both the cytoplasm and nucleus, while QKI-7 is predominantly found in the steady-state cytoplasm (Pilotte et al., 2001). Both QKI-6 and QKI-7 mainly function in the cytoplasm, responsible for the transport of target RNA and participate in stability regulation, promoting the differentiation of oligodendrocytes (Larocque et al., 2002).

Members of the STAR protein family contain one to two heterogeneous ribonucleoprotein granule K homologous (abbreviated as KH) domains and several Src homology 3 (SH3) domains related to tyrosine phosphorylation signal transduction (Chen and Richard, 1998). Due to the high conservation of amino acid sequences in STAR proteins and their dual function of signal transduction and RNA binding, they play crucial roles in embryogenesis, tissue organ development, and the regulation of protein synthesis and expression. Moreover, QKIs can also exert functions by influencing transcription, translation, transport between the nucleus and cytoplasm, and non-coding RNA actions (Larocque et al., 2002; Wang et al., 2013; Zong et al., 2014; Conn et al., 2015). QKI plays an essential role in nerve and myelin sheath formation, regulating the differentiation of neural stem cells at various aspects of post-transcriptional level, and is indispensable for embryonic development. Several point mutations in the homozygous state within the QKI gene coding region lead to embryonic lethality at early stages (Cox et al., 1999). In the context of cardiac muscle development, QKI is involved in the formation of myofibrillar structures in cardiomyocytes by modulating the alternative splicing of mRNAs such as ACTN2 (Chen et al., 2021). It has also been recently discovered that disruptions in QKI regulation during the late stages of pregnancy may be associated with developmental abnormalities of the congenital diaphragmatic hernia lung (Miyake et al., 2024). Additionally, QKI plays diverse roles in the progression of cancers.

## 2 Principal mechanisms of QKI’s function in the nervous system

### 2.1 Role of QKI in myelination and oligodendrocytes

Within the central nervous system (CNS), oligodendrocytes are responsible for forming myelin sheaths around neuronal axons, which provide physical protection and metabolic support to axons. The QKI-6 and QKI-7 isoforms are almost completely absent in the oligodendrocytes of Qkv (The Qkv mouse) mice, leading to abnormal expression of downstream mRNAs and proteins. This disruption affects the differentiation of oligodendrocytes and consequently results in the formation of abnormal myelin sheaths. Additionally, QKI-5 is a regulatory factor for myelin sheath formation and is significantly associated with oligodendrocyte damage. QKI-6 primarily influences the formation of early myelin sheaths, and QKI-6 alone selectively rescues the expression of its high-affinity mRNA ligands, such as myelin basic protein (MBP) mRNA (Hardy et al., 1996). The role of QKI-7 is bidirectional, inducing apoptosis in fibroblasts and oligodendrocytes. The target mRNAs s of QKI include myelin basic protein (MBP), early growth response protein 2 (EGR-2), p27Kip1, hnRNP A1, microtubule-associated protein 1B (MAP1B), apoptosis-inducing protein 1 (AIP-1), and cardiotrophin. Thus, QKI appears to play a major role in development, morphogenesis, cell adhesion, cell growth and maintenance, and cell communication. For example, QKI-6 can enhance the stability of MBP mRNA (Neumann et al., 2022), while reducing the expression of AIP-1 mRNA and hnRNPA1 (Doukhanine et al., 2010), thus promoting the differentiation and maturation of oligodendrocyte precursor cells (OPCs). Additionally, the stability of two key genes in myelin sheath development, MAP1B (Zhao et al., 2006) and Sirtuin 2 (SIRT2) (Thangaraj et al., 2017), is also regulated by normal QKI function (Shaoping et al., 2011).

Furthermore, QKI plays a central regulatory role in lipid metabolism within the central nervous system. It is reported that cholesterol biosynthesis within oligodendrocytes is crucial for the formation of myelin sheaths (Smolič et al., 2021), the absence of QKI-dependent coactivation can significantly disrupt the transcription of proteins related to the cholesterol biosynthesis pathway mediated by SREBP2 (sterol regulatory element binding protein-2), leading to the destabilization of myelin and resulting in severe congenital hypomyelination (Shin et al., 2021).

### 2.2 Regulation of astrocytes by QKI

Astrocytes (often abbreviated as AS) accompany the development of neurons throughout the entire process within the CNS (Central Nervous System). Their primary functions include providing a connective scaffold for neuronal cells, offering nutritional, metabolic, and immune support to neural networks, making them critical cells in maintaining the physiological homeostasis of the CNS (Smolič et al., 2021). QKI plays a significant and intricate role in regulating astrocytes. Larocque and colleagues found a connection between QKI and the differentiation of neural progenitor cells, with progenitor cells transfected with QKI differentiating into GFAP-positive astrocytes (Larocque et al., 2005). GFAP is an acknowledged marker for astrocyte differentiation and forms the primary intermediate filament protein in mature astrocytes. In primary human astrocytes, the silencing of QKI leads to a notable decrease in GFAP mRNA levels (Radomska et al., 2013). RNA sequencing of mouse brain astrocytes has also shown significant enrichment of QKI (Sakers et al., 2017). Whole-genome RNA sequencing (RNA-seq) studies on SCZ patients have confirmed that QKI is among the genes that show significant differences in these patients (Wu et al., 2012).

Gene analysis of specific subgroups of mature astrocyte genes lost due to QKI knockout indicates an enrichment of glycoproteins, lipid metabolism, and signaling peptide genes. This suggests that QKI regulates the translation of proteins located on or near the cell surface. In the mature phase of astrocytes, QKI might dynamically modulate lysosomal functions associated with synaptic pruning in astrocytes, as well as the degradation and clearance of its products (Takeuchi et al., 2020).

## 3 Research on QKI in neurological diseases

### 3.1 Schizophrenia

The human QKI gene is located on chromosome 6q26 (Li et al., 2002). Vulnerable gene loci associated with schizophrenia (SCZ) are situated on chromosome 6q25-27 (Lindholm et al., 2001; Taştemir et al., 2006). Specifically, they are pinpointed to a 0.5 Mb region, with QKI identified as the sole gene in this area deemed of significant research value (Aberg et al., 2006). Several independent studies have indicated that QKI mRNA expression is reduced in multiple regions of the brain in SCZ patients (Aberg et al., 2006; Haroutunian et al., 2006; McCullumsmith et al., 2007). Increasing evidence suggests that dysfunction in myelin and oligodendrocytes might contribute to the progression of this disease. Proper and functional neural circuits in the brain depend on the ability of oligodendrocytes to form normal myelin. Given the essential role of QKI in oligodendrocyte and myelin developmental regulation, it might be implicated in the etiology of SCZ.

Pathological changes often seen in schizophrenia patients include white matter abnormalities and decreased mRNA expression of oligodendrocyte/myelin-associated genes (Katsel et al., 2005). These changes resemble those observed in qkv mice. Haroutunian and colleagues found reduced QKI mRNA expression in Brodmann areas 44, 46, 23, 32, 22, 36, 7, and the hippocampus in SCZ patients, suggesting that aberrant QKI expression might lead to schizophrenia by inducing anomalies in oligodendrocyte and subsequent myelin-associated gene expression (Haroutunian et al., 2006). Aberg et al. observed myelin pathological changes in the brains of SCZ patients, accompanied by a decrease in the expression of oligodendrocyte/myelin genes and levels of QKI-7 and QKI-7b splice variant mRNAs. Moreover, QKI-7 and -7b were predominantly and significantly reduced in SCZ brains (Aberg et al., 2006), implying that the QKI-7 subtype might have a greater contribution to the pathological development of SCZ. Notably, the QKI deficiency in SCZ correlates with reduced mRNA expression vital for oligodendrocyte and myelin development (Katsel et al., 2005; Aberg et al., 2006), including Myelin-Associated Glycoprotein (MAG), MBP, Proteolipid Protein 1 (PLP1), SOX10, and transferrin (Aberg et al., 2006; McCullumsmith et al., 2007). Some of these genes contain QKI binding sites (Artzt and Wu, 2010; Feng and Bankston, 2010; Morikawa and Manabe, 2010). The expression defects of QKI in schizophrenia might lead to selective splicing defects of several myelin genes. In adult mice, structural defects in the nodes and paranodal regions of axons emerge after the loss of QKI expression in oligodendrocytes. Abnormal function in neural networks is believed to be central to the etiology of schizophrenia. Thus, the lack of QKI might lead to dysfunctional domains on axons (Ranvier nodes), potentially impairing neuronal function and neurotransmitter release (Davis et al., 2003). In studies on psychotic patients, those treated with typical neuroleptics showed increased QKI mRNA levels compared to those treated with atypical neuroleptics (McInnes and Lauriat, 2006), suggesting that antipsychotic drugs can influence QKI levels. Therefore, QKI deficiency might be a potential factor in the impaired development of oligodendrocytes and myelin in SCZ.

### 3.2 Depression

QKI plays a significant role in the process of neural demyelination. Pathological changes in neural myelin are widely observed in patients with depression, indicating that the QKI protein may play a vital role in the onset and progression of depression. In microarray studies of the frontal cortex regions of brains from individuals who died by suicide, there was a significant reduction in the expression of neuroglial-specific genes, especially genes involved in myelin formation (Klempan et al., 2009). There’s a reduced expression of oligodendrocyte-related genes in the temporal pole of patients with depression (Aston et al., 2005). Moreover, studies by Aston and colleagues detected nearly identical oligodendrocyte abnormalities in both non-suicidal and suicidal groups with depression, suggesting that changes in the expression of myelin formation-related genes might be specific to depression. Post-mortem studies focusing on the neuropathology of glial cells in depression reinforced this concept, although findings remain inconsistent in various cases. There’s a pronounced decrease in the expression, at both the protein and mRNA levels, of multiple QKI protein subtypes in the cortical, hippocampal, and amygdala regions of individuals with severe depression who died by suicide (Klempan et al., 2009). Currently, comprehensive research regarding the regulatory function of QKI in the onset and development of depression within the central nervous system, as well as its underlying mechanisms, has not been conducted. This could become a novel direction for future QKI functional research.

### 3.3 Ataxia

A characteristic feature of many human hereditary neurodegenerative diseases is the degeneration of Purkinje cells (PC). In an analysis of 54 proteins from 23 hereditary forms of ataxia in Purkinje cells, a total of 29 proteins were identified that interact with QKI. Many proteins that cause ataxia interact with each other and share binding regions. Although these ataxic diseases are caused by different gene mutations, they share common protein interactions and pathways (Lim et al., 2006). The qkv mice are categorized alongside other autosomal recessive hereditary human ataxias. Both qkv and qke5 mice exhibit swelling of Purkinje cell axons, suggesting neuronal degeneration (Suzuki and Zagoren, 1975; Noveroske et al., 2005). Moreover, the myelin levels in the central and peripheral nervous systems (CNS and PNS) of qkv mice are significantly reduced, emphasizing QKI’s vital role therein. From this, it can be inferred that there may be a link between QKI and ataxia. Additionally, the three QKI subtypes – QKI-5, QKI-6, and QKI-7 – all possess a common KH domain. Studies have found that mutations in other proteins with the KH domain are associated with ataxia-like syndromes, such as Nova (Yang et al., 1998) and FMRP (Hagerman, 2006).

### 3.4 Alzheimer’s disease

Neuroglial cells play a central role in brain physiology, primarily maintaining the homeostasis of the brain. The most common pathological feature of AD is the accumulation of abnormal protein aggregates, such as the abnormal deposition of β-amyloid (Aβ) plaques in the brain, leading to neuronal death. Other pathological features include morphological changes in neuroglial cells, especially the hypertrophy and reactivity of astrocytes. Reactivity is a pathological marker of astrocytes responding to injury or toxic molecules (Ju et al., 2022). The progression of AD is characterized by astrocytes transitioning from a neuroprotective state to a state of reactive gliosis (Canning et al., 1993), which prevents axonal growth and reduces neuronal inhibition (Ortinski et al., 2010). The activation of astrocytes and microglia is associated with the formation of β-amyloid aggregates (Orre et al., 2014). GFAP, a protein essential for astrocyte proliferation, has been found to be upregulated in human AD brain tissues (Kamphuis et al., 2014). GFAP is associated with the activation of astrocytes and is regulated by QKI. Moreover, the density of astrocytes and GFAP expression levels correlate with the severity of the Braak stage (Wharton et al., 2009), suggesting a link between astrogliopathy and the severity of AD. Myelin basic protein (MBP), a component of oligodendrocyte and Schwann cell myelin formation, co-localizes with amyloid in the brains of AD patients and increases in AD, possibly due to axonal injury (Zhan et al., 2015). Furthermore, MBP has been shown to interfere with the formation of β-amyloid precursor (Hoos et al., 2009). All subtypes, QKI5, QKI6, and QKI7, are upregulated in AD samples, and the expression of QKI increases with the severity of the AD state (Gómez Ravetti et al., 2010; Farnsworth et al., 2016). However, analysis by Feng and colleagues suggests that QKI may have expression differences in the early stages of AD, but not in the later stages (Feng et al., 2014). Still, any link between QKI expression and AD-related genes is currently speculative, and whether QKI is involved in the pathogenesis of AD needs further elucidation.

## 4 Research on QKI in malignant tumors

### 4.1 QKI’s tumor suppressive function in brain tumors

Gliomas originate from the glial cell lineage, primarily astrocytes, oligodendrocytes, and ependymal cells. Surgical resection, radiation therapy, chemotherapy, and temozolomide (TMZ) treatment remain the gold standard for managing GBM. However, the average survival time for GBM patients is less than 1 year, with the majority at risk of recurrence except for a few long-term survivors. In about 30% (6/20) of human glioblastomas, QKI expression has been altered, whereas in neurofibromas and meningiomas, QKI expression remained unchanged (Li et al., 2002). Subsequent studies on astrocytomas and GBM identified deletions in the 6q25-26 region, primarily involving QKI and PACRG genes (Ichimura et al., 2006; Mulholland et al., 2006). Angiocentric glioma (AG) is a rare subtype of neuroepithelial tumor in children and young adults, characterized by a vascular-centric and slender star-shaped cellular morphology. It often presents with epileptic seizures and has been identified with MYB locus deletions (Roth et al., 2014). Zhang et al. reported MYB rearrangements in AG (Zhang et al., 2013), followed by Bandopadhayay and colleagues confirming MYB-QKI rearrangements in AG (Bandopadhayay et al., 2016). MYB-QKI fusions were also found in pediatric low-grade gliomas (pLGG) (Ramkissoon et al., 2013). The breakpoint of the MYB-QKI rearrangement occurs in intron 4 of QKI, while MYB occurs between introns 9–15. Of the 147 non-angiocentric gliomas analyzed by WGS or RNA-seq, none exhibited MYB-QKI fusions, suggesting that this fusion might be a specific target for AG (Bandopadhayay et al., 2016) (Table 1). However, MYB-QKI rearrangements are not common in all GBMs (Ramkissoon et al., 2013), with most GBMs primarily presenting p53 mutations (Ichimura et al., 2006), EGFR amplifications and/or rearrangements (Liu et al., 2000), and PTEN deletions (Knobbe and Reifenberger, 2003). The QKI locus is a common fragile site, characterized by significant genomic instability and resulting frequently mutations of various cancers (Smith et al., 2006). QKI and PARK2 are located within the same minimal common region on chromosome 6q26-27; PARK2 (Veeriah et al., 2010) is a tumor suppressor gene in GBM (glioblastoma), and has an additive effect with QKI in tumor suppression. Therefore, the dual inactivation of PARK2 and QKI may collaboratively drive the development of GBM (Chen et al., 2012).

**Table 1:** QKI related variants in AG.

References	No.	Site	Genetics	Op and adjuvant Tx	Outcome
Qaddoumi et al. (2016)	6	Parietal	*MYB::QKI* fusion	NM	NM
	7	Parietal	*MYB::QKI* fusion	NM	NM
	8	Frontal	*MYB::QKI* fusion	NM	NM
	9	Temporal	*MYB::QKI* fusion	NM	NM
	10	Temporal	*MYB::QKI + BRAF* V600E	NM	NM
	11	Temporal	*MYB::QKI* fusion	NM	NM
	12	Frontoparietal	*MYB::QKI* fusion	NM	NM
	13	Temporal	*MYB::QKI* fusion	NM	NM
	14	Parietal	*MYB::QKI* fusion	NM	NM
	15	Temporal	*MYB::QKI* fusion	NM	NM
	16	Occipital	*MYB::QKI + BRAF* V600E	NM	NM
	17	Frontal	*MYB::QKI* fusion	NM	NM
	18	Temporal	*MYB::QKI* fusion	NM	NM
	19	Temporal	*MYB::QKI* fusion	NM	NM
	20	Temporal	QKI	NM	NM
Bandopadhayay et al. (2016)	21	NM	*MYB::QKI* fusion	NM	NM
	22	NM	*MYB::QKI* fusion	NM	NM
	23	NM	*MYB::QKI* fusion	NM	NM
	24	NM	*MYB::QKI* fusion	NM	NM
	25	NM	*MYB::QKI* fusion	NM	NM
	26	NM	*MYB::QKI* fusion	NM	NM
	27	NM	Other *MYB* mutation	NM	NM
Chan et al. (2017)	28	Inferior Pons	*MYB::QKI* fusion	Biopsy only	NM
D’Aronco et al. (2017)	29	Pons and medulla	*MYB::QKI* fusion	Biopsy only, Unresectable, Carboplatin, and Vincristine + Bevacizumab	Initially progressed but stable size in 12 months
	30	Brainstem	*MYB::QKI* fusion	Biopsy only, Unresectable, carboplatin, and vincristine + Bevacizumab, mTOR inhibitor	Initially progressed but stable in 4 years
Lake et al. (2020)	31	Thalamus	*MYB::QKI* fusion	Radiotherapy	NM
	32	Lt frontal	*MYB::QKI* fusion	Chemotherapy	NM
Suh et al. (2023)	33	Lt frontal	*MYB::QKI* fusion	CCRT + trial	One recurs 20 months after surgery and no further recurs 40 months after the initial surgery
Suh et al. (2023)	34	Rt hemisphere CC and Thalamus	*MYBL1::QKI* fusion		

QKI primarily functions as a tumor suppressor gene in GBM. Previous studies have identified downregulation of QKI in a subset of GBM patients. QKI is a downstream target of p53, which is the most frequently mutated tumor suppressor gene in primary GBM. p53 directly activates QKI, and influencing relative genes and pathways (Chen et al., 2012). Pten controls the renewal and differentiation of neural and glioma stem cells in synergy with P53. The concurrent specific deletion of p53 and Pten in central nervous system of mice could lead to high-grade malignant glioma phenotypes which closely resemble human primary GBM in clinical, pathology, and molecular characteristics (Zheng et al., 2008). Subsequently, Takashi Shingu and colleagues established the first mouse model of QKI-deficient GBM, demonstrating that the co-deletion of Pten, p53, and QKI in mice NSCs (neural stem cells) resulted in 92% malignant glial tumors, which closely resemble human glioblastoma in terms of morphology, metastasis, proliferation, and heterogeneity. The QKI protein is involved in RNA processing and splicing, participating in the regulation of protein translation and other processes, and the absence of QKI gene indicates a decrease in endosome and lysosomal generation ability. Neural stem cells normally maintain the stemness only in the subventricular zone (SVZ), however, the loss of QKI significantly elevates the levels of receptors necessary for maintaining stemness on the cell membrane, allowing them to preserve stem cell characteristics even after leaving SVZ (Shingu et al., 2017). Thus, QKI has the function of reducing the stemness of tumor cells, and the loss of QKI may affect downstream RNA targets involved in carcinogenesis, transformation, or angiogenesis, potentially being an important condition for the progression of GBMs.

QKI can regulate RNA homeostasis in multiple aspects, such as RNA stability, alternative splicing, translation, miRNAs, and circular RNAs. miRNAs are small non-coding RNAs that bind to specific mRNAs and regulate their translation and/or stability, and are associated with the pathogenesis in nearly all known cancer pathways. QKI has been shown to influence the stability of downstream miR-20a, thereby suppressing the TGFβR2 signaling pathway. This regulatory effect has been substantiated both *in vitro* and *in vivo* using primary mouse astrocytes and human glial cells, demonstrating its impact at the molecular, biochemical, and functional levels. Moreover, all three common isoforms of QKI are implicated in the modulation of miR-20a (Chen et al., 2012).

miR-29a has been identified to directly repress the expression of QKI-6, with WTAP (Wilms’ tumor 1-associated protein) serving as its downstream target, which inhibit the phosphoinositide 3-kinase/AKT and extracellular signal-regulated kinase pathways (Xi et al., 2017). Furthermore, an increased expression of miR-148a has been observed in GBM glioma cell lines, which targets and diminishes the expression of QKI, leading to the augmented and sustained activation of NF-κB and TGF-β/Smad signaling pathways, which in turn inhibits cell proliferation, migration, and invasion.

The role of QKI in malignant tumors is garnering increasing attention. Consequently, elucidating the regulatory network and mechanism of QKI, including its upstream and downstream effectors, is instrumental in exploring its potential value in the diagnosis and treatment of malignant tumors.

### 4.2 Regulatory mechanisms of QKI in other malignant tumors

In brain tumors, previous research mainly focused on AG subset, characterized with MYB-QKI rearrangement primarily. In GBM, only WTAP, SHH, GLI1, NF-κB, and TGF-β pathways have been identified. We also found that QKI is involved in the regulation of multiple miRNAs, a type of non-coding RNAs. The potential influence of QKI on other non-coding RNAs remains to be determined, therefore, we attempted to find direction by investigating the mechanism of action of QKI in other tumors.

#### 4.2.1 QKI and non-coding RNA in tumors

QKI is closely associated with the occurrence and progression of various cancers, including lung, gastric, and prostate cancer, etc. As a tumor suppressor in multiple malignancies, QKI is increasingly recognized as a significant biomarker for evaluating tumor progression and prognosis. Numerous studies have indicated that QKI can modulate tumor growth by enhancing the stability of non-coding RNAs, selective splicing, and cellular cycle regulation, and it influences epithelial-mesenchymal transition (EMT) to promote tumor metastasis. Furthermore, QKI is subject to methylation of its promoter region (de Miguel et al., 2016) and the impact of the tumor microenvironment. Non-coding RNAs, which mainly include microRNA (miRNA), long non-coding RNA (lncRNA), and circular RNA (circRNA), play important roles in the initiation and development of cancer.

The QKI protein possesses the function of regulating the formation and activity of miRNAs and also can regulated by miRNAs, forming a regulatory network. QKI can influence the cell cycle, migration, and proliferation by modulating the expression of miRNAs, for example, QKI-5 regulates miR-196b-5p (Liang et al., 2020) and miR-31 (Zhu et al., 2023). In most instances, QKI serves as a target gene for miRNAs, including miR-155, miR-221, miR-200c, miR-200, miR-574-5p, miR-143-3p (He et al., 2016), and miR-362-5p (Wei et al., 2020), which mostly are associated with tumor proliferation and migration. miR-155 and miR-221 can regulate the cell cycle and invasive capacity of colon cancer by reducing the production of QKI (He et al., 2015; Mukohyama et al., 2019), while miR-574-5p inhibits the expression of QKI, particularly QKI-6/7, increasing proliferation, migration, and invasion, and decreasing differentiation and cell cycle arrest (Ji et al., 2013).

The expression of QKI is also related to tumor angiogenesis. It is reported that the miR-200 family inhibits tumor cell migration by regulating tumor angiogenesis. miR-200 blocks the expression of QKI in tumor endothelial cells, leading to increased production of QKI, thereby promoting angiogenesis and nutrient supply (Azam et al., 2019). Moreover, QKI is involved in tumor metastasis. miR-574-5p targets QKI to inhibit the growth and metastasis of cervical cancer cells and enhances chemosensitivity (Ji et al., 2013; Tong et al., 2020). By regulating miR-200c, QKI-5 inhibits EMT and the invasion and migration of renal clear cell carcinoma. Upregulation of QKI-5 also increases the EMT related proteins, including vimentin, snail, and slug proteins, while downregulating E-cadherin, promoting cell migration and invasion (Zhang et al., 2021).

Circular RNAs (circRNA) are recently discovered non-coding RNA molecules derived from the back-splicing of precursor mRNA. These are endogenous closed-loop RNAs. Their circular form confers resistance to nuclease degradation, making them more stable than linear RNAs. Aberrant expression of circRNA influences various biological mechanisms such as gene transcription regulation, acting as miRNA sponges, involvement in protein translation, and interactions with RBPs (Du et al., 2017; Xu et al., 2018). Studies have shown that they can serve as pivotal regulators in the cancer cell cycle, apoptosis, proliferation, invasion, and migration (Chen et al., 2020). The biogenesis of circRNAs is modulated by exon-skipping events and RNA binding proteins (RBPs). These RBPs include ESRP1, ESRP2, PTBP1, TNPO1, RBM, and QKI, which function as splicing factors modulating selective splicing (Jeck and Sharpless, 2014). Quaking (QKI) belongs to the STAR family with KH domains and affects the splicing of pre-mRNA. Introducing common binding sequences of QKI into flanking introns leads to the formation of circRNAs from exons that are typically subjected to standard linear splicing. Due to the dimerizing capability of QKI, it is believed to target flanking introns, bringing circularized exons closer together, thereby enhancing the generation of circRNAs (Conn et al., 2015).

In NSCLC, QKI can directly bind to the flanking introns of circ-SLC 7A6, promoting the production of circ-SLC7A6, which inhibits proliferation and invasion. Furthermore, miR-21 is a direct functional target of circ-SLC7A6 (Wang et al., 2020). Although QKI facilitates circRNA formation, some studies found that QKI expression is regulated by circRNAs. For instance, circ-UBR5 can bind to the KH domain of QKI (Qin et al., 2018), circ-MTO1 acts as a sponge for oncogenic miR-17, promoting QKI-5 expression, leading to the inactivation of the Notch signaling pathway, thus inhibiting LUAD growth. Similarly, circSHPRH acts as a sponge for miR-224-5p, regulating QKI expression, significantly suppressing the proliferation, EMT, invasion, and migration of human bronchial epithelial BEAS-2B cells (Zhou et al., 2021). The G-protein-coupled estrogen receptor (GPER) can also promote NSCLC cell growth through the YAP1/QKI/circNOTCH1/m6A methylation of NOTCH1 pathway (Shen et al., 2021). Recent research suggests that in many instances, circRNAs do not act as miRNA sponges (Guo et al., 2014). For example, while QKI promotes the formation of circNDUVB2, inhibiting the progression of NSCLC (Li et al., 2021), circNDUFB2 does not function as a miRNA sponge in the studies. Downregulation of QKI-5 is associated with the decreasion of circZKSCAN1 (Zhu et al., 2019), and is involved in the generation of circGSK3B (Li et al., 2020). Additionally, QKI serves as a miR-1265 sponge, positively regulating CAB39, which promotes the reprogramming of glutamine metabolism. High expression of QKI facilitates alternative splicing and the production of circRNAs, leading to an RNA storm that upregulates genes related to proliferation, migration, and angiogenesis, thereby enhancing the malignancy of liver cancer (Han et al., 2019). Furthermore, circSPIRE1, circARFGEF2, circBCAR3, circRNA-SFMBT2, and circSLC26A4 are all positively regulated by QKI through a feedback mechanism (Ji et al., 2020; Xi et al., 2022; Kong et al., 2023; Li et al., 2023; Shu et al., 2023).

Long non-coding RNAs (lncRNAs) are defined as RNAs longer than 200 nucleotides that do not code for proteins. Similar to mRNAs, they are transcribed by RNA polymerase II. However, in contrast to mRNAs, many lncRNAs are preferentially located in the cell nucleus. They perform a multitude of functions, including nuclear functions such as regulating gene expression in cis or trans, splicing modulation, and nucleation of sub-nuclear domains (Goodall and Wickramasinghe, 2021).

**Figure 1:**
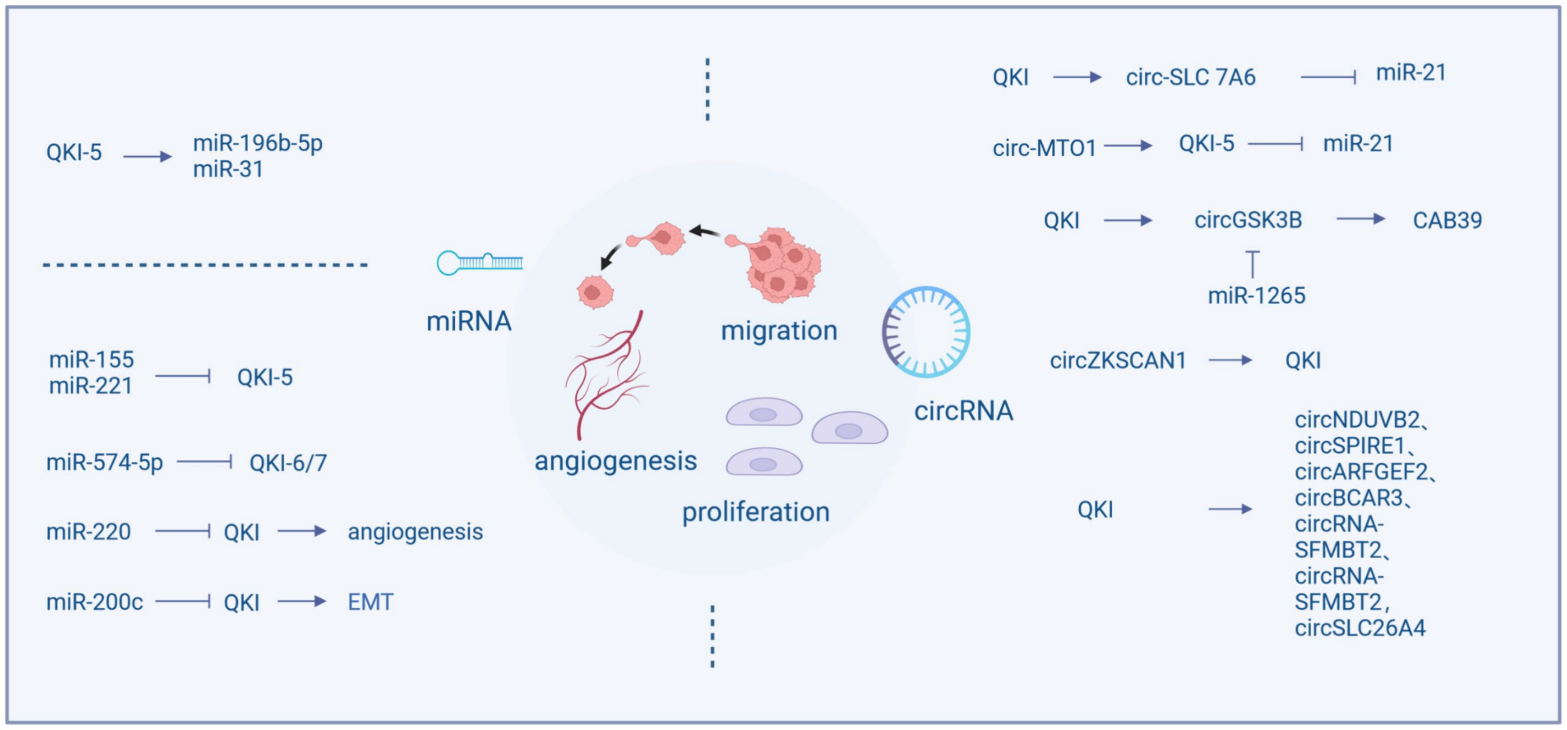
Regulation of QKI.

LncRNAs can act as oncogenes. LncRNA-MEG3 affects the ability of cell proliferation, migration, and invasion, as well as the rate of cell apoptosis. This regulation is dependent on miR-9-5p and QKI-5 (Wu et al., 2019). LncRNAs can also function as tumor suppressor genes. LncRNA TPT1-AS1 is involved in the development of various cancers. In breast cancer, TPT1-AS1 and QKI share a binding site in miR-330-3p. A low expression of TPT1-AS1 is significantly associated with some clinical features of malignant tumors, such as high TNM staging, lymph node metastasis, Her-2 negative status, and a shorter overall survival (Hu et al., 2020) (Figure 1).

Thus, it is evident that QKI plays a p crucial role in the regulation of non-coding RNA in other types of tumors. Moreover, miRNAs, lncRNAs, and circRNAs interact with each other and are dependent on QKI’s regulation. QKI may act as an upstream or downstream component within this network, which provides a reference for our research into the mechanisms of brain tumorigenesis. For instance, we can investigate whether it has the same functional pathways in brain tumors and whether it can serve as a target for therapy and prevention.

### 4.3 Methylation of QKI

Research has discovered that the low expression of QKI in various tumors may be related to the methylation of its promoter, with this methylation facilitating the inactivation of QKI. In colorectal cancer (CRC), QKI is significantly downregulated, to the point of being absent, in part due to hypermethylation of its promoter. Studies have identified that high methylation of the QKI promoter is the reason for decreased QKI expression in colorectal cancer, stomach cancer, and prostate cancer (Flores et al., 2008). In CRC, methylation of the QKI promoter was detected in 32.1% of CRC patients. Compared to matched normal tissues, these patients had significantly reduced QKI mRNA expression in their tumor tissues, with a notable correlation between low QKI expression and postoperative recurrence (Iwata et al., 2017). Furthermore, QKI manifests as highly methylated in the cfDNA of CRC patients, suggesting it might serve as a CRC-specific methylation biomarker (Zhang et al., 2022). Overexpression of QKI in CRC cells elevates the expression of intestinal differentiation markers, intestinal alkaline phosphatase, and lactase, concurrently increasing the protein expression levels of p27Kip1 (Yang et al., 2010). In lung cancer, hypermethylation of the QKI-5 promoter leads to reduced expression and inhibits EMT induced by TGF-β1 (Zhou et al., 2017). It is not yet known whether QKI is regulated by methylation in brain tumors. Moreover, MGMT promoter methylation is an important predictive biomarker for chemosensitivity to alkylating agents and patient prognosis in GBM. Therefore, whether QKI methylation could serve as an additional biomarker is also worth exploring and investigating.

### 4.4 QKI and cancer treatment

QKI plays a regulatory role in multiple aspects of tumor progression, including tumor growth, metastasis, and angiogenesis, and is associated with prognosis. Targeting QKI can serve as a tool to counteract the stemness, invasiveness, angiogenesis, and treatment resistance of tumors, benefiting the disease. Reports indicate that QKI-5 can modulate cicZKSCAN1 and influence hepatocellular carcinoma (HCC) cells treated with sorafenib (Song et al., 2023). The YY1 binds to the super-enhancer and promoter of QKI, leading to its abnormal activation, subsequently inducing the formation of circRNAs in HCC and triggering epithelial-mesenchymal transition (EMT) and tumor metastasis. Hyperoside, one of the flavonol glycoside compounds derived from several plants, can target the YY1/p65/p300 interaction, obstructing the assembly of this complex. This hinders the binding to the super-enhancer, suppressing QKI expression and exerting anti-liver cancer effects (Han et al., 2019). A recent study demonstrated that the combined therapy of radiation (RT) and metformin suppresses the progression of prostate cancer (PCa) by modulating the QKI/circZEB1/miR-141-3p/ZEB1 signaling pathway, enhancing radiation sensitivity. Additionally, metformin-induced AMPK signaling converges at FOXO3 to stimulate SETD2 expression, subsequently enhancing its substrate EZH2’s enhancer to delay PCa metastasis. The combination of metformin and the EZH2 inhibitor GSK126 synergistically inhibits PCa cell growth *in vitro* and *in vivo*, affirming metformin as a promising therapeutic approach for future prostate cancer treatment (Chen et al., 2020).

A primary reason for cancer treatment failure is the acquisition of drug resistance. CCAT1 promotes HCC proliferation and reduces cell apoptosis induced by oxaliplatin. Knocking out CCAT1 can enhance chemical sensitivity both *in vitro* and *in vivo*. Further studies revealed that QKI-5 is a crucial mediator, and blocking the QKI-5/p38MAPK signaling pathway can enhance the sensitivity to oxaliplatin (Xia et al., 2022). In ER+ breast cancer, QKI promotes the formation of circRNA-SFMBT2. Elevated expression levels of circRNA-SFMBT2 promote cell growth and tamoxifen resistance (Li et al., 2023). Moreover, QKI and circRNA participate in cellular irradiation responses. After ionizing radiation, the transcription of QKI and its interaction with KIRKOS-71 and KIRKOS-73 significantly increases (O’Leary et al., 2017).

The role of immune modulation in the development of malignant tumors cannot be underestimated, and recently, the significance of immunotherapy in cancer treatment has become increasingly prominent. The QKI protein can exert anti-tumor effects through immune regulation. Studies have reported that the knock-out of QKI can upregulate genes induced by interferons, suggesting that QKI may act as an immune suppressor (Liao et al., 2021). Overexpression of QKI can increase the production of the anti-inflammatory cytokine IL-10. Macrophages, as key players in innate immunity and the primary cells initiating inflammation, develop into tumor-associated macrophages (TAMs) within tumors. QKI can dynamically regulate the polarization state of macrophages and function in inhibiting innate immune responses by modulating the NF-κB pathway (Wang et al., 2017). In early differentiated monocyte progenitor cells, transcriptional activation of QKI-5 can downregulate the expression of CSF1R, thereby negatively regulating macrophage differentiation and forming a negative feedback loop during the macrophage differentiation process (Fu et al., 2012). Therefore, QKI may influence tumor immune modulation by participating in inflammation. Additionally, QKI is associated with the prognosis of diffuse large B-cell lymphoma (DLBCL) (Pan et al., 2021), NSCLC (Li et al., 2021), and leukemia (Tili et al., 2015). In summary, research has revealed that QKI plays a critical role in angiogenesis, apoptosis, cell growth, and immunity, and it may become a novel biomarker for diagnosis, treatment, and evaluation in brain tumors. The development of targeted or immunotherapeutic drugs related to QKI is a promising direction; however, the signaling pathways and molecular mechanisms involved are not yet fully understood and require substantial basic and clinical trial support for further research and evaluation.

## 5 Conclusion

**Figure 2:**
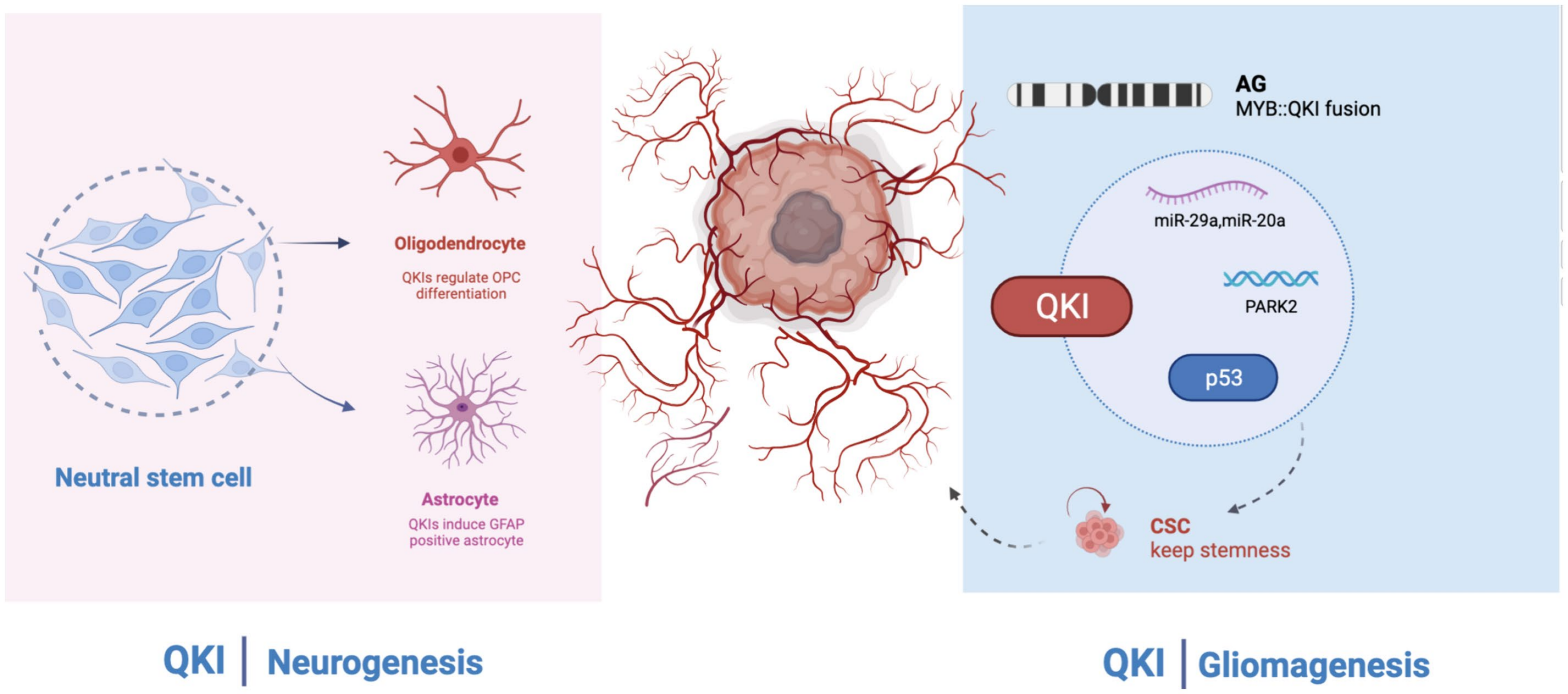
QKI regulates neural development and glioma formation. Left: QKI regulates oligodendroglial cells and astrocytes; Right: In GBM (Glioblastoma Multiforme), QKI acts as a tumor suppressor gene and is involved in multiple signaling pathways. MYB: QKI fusion is commonly observed in AG (Astrocytoma Grade).

QKI has long been believed to participate in the differentiation, development, and regulation of the central nervous system, playing a crucial role in processes like alternative splicing, the stability and localization of mRNA, and non-coding RNA, as well as mRNA translation. The association of QKI protein with human pathology suggests that in the central nervous system, QKI is involved in the onset of various neuropsychiatric disorders, including schizophrenia, depression, ataxia, and Alzheimer’s disease. However, its core regulatory functions and specific mechanisms have not been systematically elucidated. Research on QKI has revealed its pivotal functions in angiogenesis, cell apoptosis, and cell growth. Its mechanism primarily involves participation in the formation or stability of non-coding RNAs, especially miRNAs and circRNAs, further affecting the prognosis of cancer patients. Simultaneously, QKI might also be regulated in return, influencing downstream signaling. It has been found that low expression of QKI appears in various tumors and might be controlled by its promoter methylation. Methylation of the promoter promotes the inactivation of QKI. The outcomes of immunotherapy in cancer treatment in recent years have been very promising. QKI has also been involved in immune-related pathways. Some anti-cancer drugs, as well as radiotherapy treatment methods, also involve QKI. Hence, QKI may potentially become a new biomarker for the diagnosis, treatment, and evaluation of malignant tumors in the future. The MYB-QKI fusion in GBM mainly occurs in angiogenic gliomas, primarily affecting the stemness of glioblastomas. The signaling pathways and signaling molecules involved in QKI’s participation in tumorigenesis have not been fully studied. QKI affects the resistance to chemotherapy and some targeted drugs, but its influence in tumors is relatively superficial, and its regulatory mechanism remains unclear. Although the direct development of small molecule inhibitors targeting MYB is challenging, targeting MYB-QKI, such as KIT or CDK6, might be a promising avenue for drug development. Additionally, MYB-QKI can bind to H3K27ac enhancer elements. Indirect inhibition of MYB-QKI, possibly through BET inhibitors or CDK7 suppression, might also play a role in GBM treatment. Further research requires the support of extensive basic and clinical trials (Figure 2).
